# Prevalence and predictors of acute respiratory infection among children under-five years in Tigray regional state, northern Ethiopia: a cross sectional study

**DOI:** 10.1186/s12879-023-08701-2

**Published:** 2023-10-30

**Authors:** Gebru Gebremeskel Gebrerufael, Bsrat Tesfay Hagos

**Affiliations:** 1 Department of Statistics, College of Natural and Computational Science, Adigrat University, Adigrat, Ethiopia; 2 Department of Statistics, College of Natural and Computational Science, Mekelle University, Mekelle, Ethiopia

**Keywords:** Children, Acute respiratory infection, Tigray regional state, Predictor factors

## Abstract

**Background:**

Acute respiratory infection is still one of the leading causes of child morbidity and mortality worldwide. Developing countries, especially in Sub-Saharan Africa including Ethiopia continue to share an overburden of this infection. Studies showed that different predictor factors were associated with the occurrence of childhood acute respiratory infection. Therefore, the main aim of this study was to assess the prevalence and associated predictor factors of acute respiratory infection among children under-five years in the Tigray Regional State, northern Ethiopia.

**Methods:**

A retrospective cross-sectional study design was done from January 18, 2016, to June 27, 2016. A total of 986 children under-five years were selected for this study. The logistic regression model analysis was employed to examine the predictor factors of childhood acute respiratory infection. Both bi-variable and multivariable data analysis was performed using STATA version 14.0.

**Results:**

Overall, the study showed that the two weeks prevalence of acute respiratory infection among children under-five years was 16.10% [95%CI: 13.80–18.40]. According to the multivariable logistic regression model analysis, children aged (24–60) months (AOR: 0.59, 95%CI: 0.352–0.98), rich wealth index of households (AOR: 0.60, 95%CI: 0.378–0.959), diarrhea status of children (AOR: 3, 95%CI: 1.97–4.73), and mothers smoking cigarettes (AOR: 4, 95%CI: 1.15–16.50), were significant predictors of acute respiratory infection.

**Conclusion:**

The prevalence of ARI displays that Tigray regional state was experiencing a higher ARI rate than the national level. The current study identified the low wealth index of households, children aged (24–60 months), mothers smoking cigarettes, and diarrhea status of the children as crucial predictor factors for ARI. Interventions should be improved to these modifiable major predictor factors that significantly decrease the ARI problem among under-five children.

**Supplementary Information:**

The online version contains supplementary material available at 10.1186/s12879-023-08701-2.

## Background

Acute respiratory infection (ARI) is the occurrence of a cough accompanied by short, rapid breathing which may be linked to death, although a significant reduction has been achieved over the previous two decades [1, 2]. Globally, ARI among children remains one of the leading causes of morbidity and mortality in children under-five years [3–5]. It is the largest public health burden worldwide, developing countries, especially in Sub-Saharan Africa (SSA) including Ethiopia have continued to share an overburden of this infection [1, 4, 6–8].

The illness burden due to ARI is 10–50 times higher in developing countries compared to developed countries [9]. Even if it occurs at all stages of the different ages; however, it is more vulnerable in under-five year’s children [2, 8, 10, 11].

ARI is one of the most health problems that should be emphasized in children under the age of five years in Ethiopia [9]. In Ethiopia, 88 in 1,000 children under five years old die before their fifth birthday due to ARI and 3 out of 10 of these children sought medical treatment [5].

In Ethiopia, there is a significant regional variation of ARI in children under-five years. According to the 2016 Ethiopia Demographic and Health Surveys (EDHS) report, the Tigray regional state is still experiencing a higher ARI rate of 77 per 1,000 total live births, compared to the national average rate of 66 per 1,000 total live births [5].

Evidence from different literature indicated that diarrhea status, low level of maternal education, sex of the child, child’s age, mother’s smoking cigarette, poor level of wealth index, and household size, and other factors, are among the predictor factors associated with childhood ARI [2, 6, 12–16].

Even though Ethiopia has made a substantial investment to reduce the morbidity and mortality of ARI; but, ARI still remains very high in the country.

Moreover, although the fact that several of investigators have been made on the identification of predictors that are related to ARI in country level, there are shortage of studies that focus on the ARI and its determining factor. The majority of these studies are focused on the countrywide level. Such studies miss an essential point of policy designers and makers as the countrywide result may not display the exact condition at regional state levels.

To address such a gap, considerable progress in reducing childhood ARI is crucial to achieve the country’s goal in the future. We conducted a holistic retrospective cross-sectional study analysis using the current 2016 EDHS, to identify the major risk factors of ARI in the Tigray regional state, Ethiopia [5].

The main aim of this investigation was to assess the prevalence and associated predictor factors of ARI in children under-five years in the 2 weeks preceding the survey in the Tigray regional state, Ethiopia.

The investigation is valuable for indirectly assessing the effectiveness of interventions aimed at childhood acute respiratory infection which may be possible to eradicate ARI caused by specific microbes using a vaccine. The investigation is also expected to help health professionals and policymakers redesign childhood acute respiratory infection control and elimination strategies.

## Methods

### Study design and period

A retrospective cross-sectional study design was done from January 18, 2016, to June 27, 2016. The study used the Tigray Regional State DHS data set. The fourth survey was conducted nationally by the Central Statistics Agency (CSA), the Ministry of Health and the Ethiopian public health institute.

This dataset was found from the 2016 EDHS survey measure program with authorization [5].

Tigray Regional State is located between 36- and 40-degrees’ east longitude north of Ethiopia bounded by the south Amhara regional state, in the east Afar regional state, the Sudan in the west and the north with Eritrea. According to the 2007 Ethiopian population and housing censuses, the Tigray regional state had a population size of 3,136,267 of which 1,542,165 were males. The majority (85%) of the population are rural residents [17].

### Source of population and study population

The source of the population was all under-five years of age children in the Tigray regional state. The study population was all sampled under-five years of age children who live in the Tigray regional state.

### Sample size and sampling methods

**Fig. 1:**
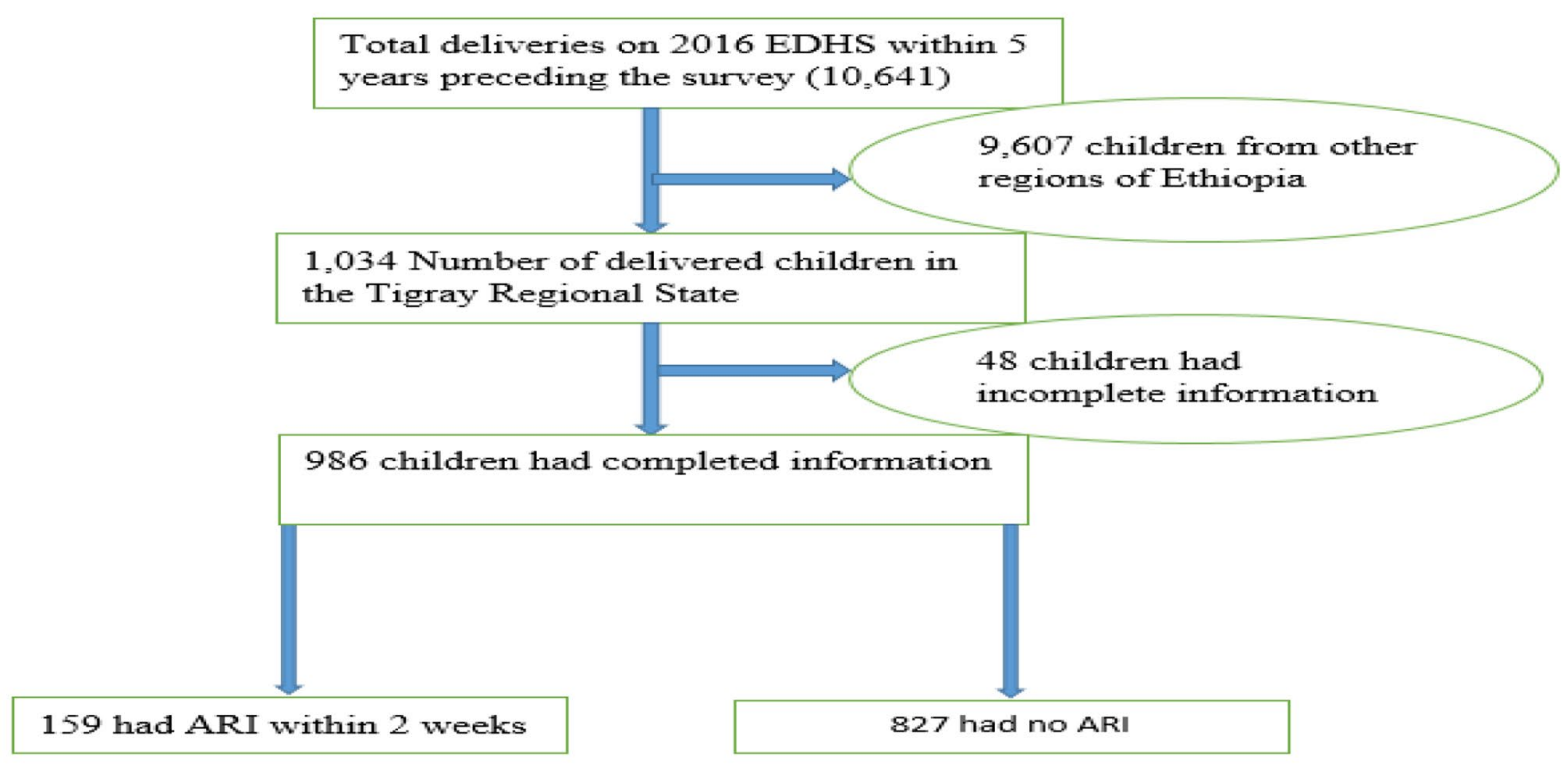
Schematic presentation of children under-five with ARI included in the study

The survey had information on a range of socioeconomic and demographic predictor factors of the population nationwide. Tigray regional state was selected because the 2016 EDHS report revealed that it had the second highest rates of ARI in the country. The samples were designated using a two-stage stratified cluster sampling method on the 2016 EDHS for Tigray regional state. Firstly, the region was stratified into urban and rural clusters. A total of 49 rural and 14 urban clusters and 381 households from Tigray region state were considered. All women aged (15–49 years) who had at least one child in the five years before the two weeks preceding the survey were eligible for participation in each cluster. In the region, 1,034 births were reported in the previous five years preceding the 2016 EDHS survey conducted. Next, children with detailed information on ARI during the last five years were presented in the full 2016 EDHS report [5]. Finally, a total of 986 children under-five have complete information about all the predictor factors considered included. Therefore, the sample size for this study was 986 children under five years were taken for the final analysis [see Fig. 1].

### Data collection procedures

Data were extracted from the 2016 EDHS children’s data set. The new data set was carefully extracted from 2016 EDHS report data concerning socio-economic, demographic, health and environmental predictor factors of childhood acute respiratory infection among children under five years.

### Study variables

#### Outcome variable

The outcome variable for this study was the ARI of children under-five years which was a dichotomous (binary) outcome variable coded as “0” for No, and “1” for Yes. Such that

$${{\text{Y}}_{\text{i}}}{\text{ = }}\left\{ {_{{\text{0,}}\,{\text{if}}\,{{\text{i}}^{{\text{th}}}}\,{\text{Child's}}\,{\text{had}}\,{\text{not}}\,\,{\text{experienced}}\,{\text{with}}\,{\text{ARI}}}^{{\text{1,}}\,{\text{if}}\,{{\text{i}}^{{\text{th}}}}\,{\text{Child's}}\,{\text{had}}\,\,{\text{experienced}}\,{\text{with}}\,{\text{ARI}}}} \right.$$

ARI was determined by the public health professionals with any symptoms and signs like cough, rapid breathing, sore throat, noisy breathing, or chest in drawing, at any time in the 2 weeks preceding the survey date. The symptoms are well-matched with acute respiratory infections [5].

#### Independent variables

The independent variables included in the study were presented in Table 1.

**Table 1:** Operational definitions and categories of independent variables

Variables	Definitions and categories
Child’s age	Child’s age, in month (< 6, 6–11, 12–23, 24–60)
Sex of child	This is defined as weather the child is male or female (male, female)
Residence	Place of residence (urban, rural)
Household size	Number of family members in each household (≤ 5, > 5)
Sex of the household head	Sex of the household head (male, female)
Mother’s education	Mother’s level of education (no formal education, primary or above)
Wealth index	Based on assets and possessions of households (poor, middle, rich)
Vaccinated status	Vaccinated status of child (not vaccinated, vaccinated)
Mother’s age	Mother’s age, in years (15–24, 25–34, 35–49)
Health insurance coverage	Health insurance coverage (no, yes)
Type of birth	Type of birth (single, multiple)
Diarrhea status	History of diarrhea status on child in the past (no, yes)
Anemia status	Anemia status of child (no, yes)
Mother’s smoking cigarette	Mother’s history of ever smoked in the past time (no, yes)
Religion	Mother’s religion status (Orthodox, Muslim)
Media exposure	Media exposure (no, yes)
Size of child at birth	Size of child at birth (smaller, medium, larger)

### Controlling bias and data quality

A completed structural questionnaire was employed as a tool for the data collection process. The man’s, the household, and the woman’s questionnaires were done to collect demographic and socio-economic information and other important information. After all the questionnaires were ready in the international English language, they were translated into the mother tongue Tigrigna language. Moreover, the collected dataset was back-translated into the international English language to keep its consistency. The quality of the data was upheld by handling the missing values and testing its completeness by running frequency tables. Before the actual data collection, the quality of the novel data sets was kept through pretesting of the questionnaires in mothers’ tongue Tigrigna language and giving training for interviewers using tablet mobiles to record the response. The tablet mobiles were prepared with Bluetooth technology to allow remote electronic transfer of files (i.e., transfer of standard questionnaires from the supervisors to interviewers and also transfer of completed questionnaires from interviewers to the supervisors).

### Data processing and analysis

STATA version-14 software was used for extracting; entering, checking completeness, re-coding, cleaning, and analysis of data for this study (see Additional File 1). Both bi-variable and multivariable logistic regression model analyses were used to assess the association of various predictor factors of childhood ARI. Descriptive statistics were used to exhibit frequencies, with percentages in tables, graphs and using figures. The strength of the association of predictor factors with the dependent variable was assessed using the odds ratio with a 95% Confidence Interval (CI).

Bi-variable logistic regression model analysis was done for each independent variable with the outcome variable and those predictor variables with a p-value of < 0.25 included in the multivariable logistic regression model analysis to identify predictor factors of acute respiratory infection. Predictor variables that were significant at a p-value 0.05 level of significance and 95% CI were considered to be the predictor factors of childhood ARI.

### Assessment of parsimonious model fitness of the model

We begin here first by checking the multicollinearity test was done to assess the existence of correlation among the predictor variables. Since the VIF value for all predictor variables is < 10 there is no multicollinearity problem (see Table 2). Additionally, we checked the overall goodness of fit using the Likelihood Ratio Test (LRT) and Hosmer-Lemeshow tests. Accordingly, the LRT provided a X^2^ (11) = 54.63 and p-value < 0.0000, which would imply goodness-of-fit found for the model. Similarly, the Hosmer-Lemeshow test found the observed data was better explained by the model [X^2^ (8) = 9.59 and p-values = 0.295].

**Table 2:** Shows the multicollinearity of predictor variables

Variables	Categories	VIF
Child’s age	< 6	Ref.
	6–11	1.49
	12–23	1.96
	≥ 24	2.3
Sex of child	Male	Ref.
	Female	1.84
Mother’s education	No formal education	Ref.
	Primary and above	1.72
Wealth index	Poor	Ref.
	Middle	1.33
	Rich	1.67
Health insurance coverage	No	Ref.
	Yes	1.14
Diarrhea status	No	Ref.
	Yes	1.18
Anemia status of child	No	Ref.
	Yes	2.2
Mother’s smoking status	No	Ref.
	Yes	1.03
	**Mean VIF**	**1.62**

## Results

### Socio-economic, demographic, environmental, and health characteristics of the mothers/children

A total of 986 children under-five years were included in the study with a response rate of 95.40%. Out of 986 children, 49.5% were male and the rest 50.50% female. Nearly half (44.40%) of the mothers were 25–34 years of age with a median age of 30 years and SD of ± 6.90 years. The majority (82.80%) of the children were from rural areas while the rest, 17.20% from urban areas of the region. Of the total study participants, 87.41% didn’t have health insurance coverage and most (95%) of respondents were followers of the Orthodox religion. The median child’s age was 15 (InterQuartile Range (IQR): 0–30) months. The majority (70.3%) of the households had greater than 5 members with a median family size of 4 (IQR: 1–8).

More than half (63.11%) of the mothers were not educated, and 36.90% had primary and above education levels. The majority (83.81%) of the household’s heads were male sex in the family. Moreover, nearly half (54%) of the households were in the poor category of wealth status, 18.30% in the medium, and 27.31% in the high wealth category (Table 3).

**Table 3:** Socio-economic, demographic, health and environmental characteristics of respondents/children in the Tigray Region State, 2016 (N = 986)

Variables	Categories	Frequency (N)	Percentage (%)
Child’s age (in month)	< 6	140	14.2
	6–11	83	8.42
	12–23	218	22.1
	24–60	545	55.3
Sex of child	Male	488	49.50
	Female	498	50.50
Residence	Urban	170	17.2
	Rural	816	82.8
Household size	≤ 5	293	29.7
	> 5	693	70.3
Sex of the household head	Male	826	83.8
	Female	160	16.2
Mother’s education	No formal education	622	63.1
	Primary and above	364	36.9
Wealth index	Poor	532	54
	Middle	180	18.3
	Rich	274	27.8
Vaccinated status	Not vaccinated	322	32.7
	Vaccinated	664	67.3
Mother’s age	15–24	254	25.8
	25–34	438	44.4
	35–49	294	29.8
Health insurance coverage	No	862	87.4
	Yes	124	12.6
Type of birth	Single	967	98.1
	Multiple	19	1.93
Diarrhea status	No	861	87.3
	Yes	125	12.7
Anemia status of child	No	976	99
	Yes	10	1.01
Mother’s smoking cigarette	No	976	99
	Yes	10	1.0
Electricity usage	No	708	71.8
	Yes	278	28.2
Religion	Orthodox	937	95
	Muslim	49	5
Media exposure	No	737	74.8
	Yes	249	25.3
Size of child at birth	Smaller	239	24.2
	Medium	547	55.5
	Larger	200	20.3

### Prevalence of ARI among children under-five years in the Tigray regional state, Ethiopia

The prevalence of ARI among children under-five years was reported to be 16.10% [95%CI: 13.80–18.40] in the two-week surveillance period, of whom 66% (89/159) were males. Among children who had anemia disease, 49.06% (78/159) developed ARI (Table 4).

**Table 4:** Bi-variable and multivariable logistic regression model analysis on predictor factors of ARI in children under-five in the Tigray Regional State, Ethiopia 2016 DHS data

Variables	Categories	ARI status of child		COR (95%CI)	AOR (95%CI)
		No	Yes		
Child’s age	< 6	112	28	1.0	1.0
	6–11	71	12	0.67(0.32, 1.4)	0.52(0.229, 1.19)
	12–23	169	49	1.2(0.69, 2.0)	0.99(0.552, 1.79)
	24–60	475	70	0.6(0.36, 0.96) *	0.59(0.352, 0.98) *
Sex of child	Male	399	89	1.0	1.0
	Female	428	70	0.73(0.52, 1.0)	0.74(0.520, 1.06)
Mother’s education	No formal education	515	107	1.0	1.0
	Primary and above	312	52	0.8(0.56, 1.1)	0.92(0.622, 1.37)
Wealth index	Poor	431	101	1.0	1.0
	Middle	154	26	0.7(0.45, 1.2)	0.70(0.429, 1.14)
	Rich	242	32	0.6(0.37, 0.87) *	0.6(0.378, 0.959) *
Health insurance coverage	No	718	144	1.0	1.0
	Yes	109	15	0.7(0.38, 1.2)	0.76(0.423, 1.36)
Diarrhea status	No	745	116	1.0	1.0
	Yes	82	43	3.4(2.2, 5.1)	3(1.97, 4.73) *
Anemia status of child	No	465	81	1.0	1.0
	Yes	362	78	1.2(0.88, 1.7)	1.1(0.736, 1.63)
Mother’s smoking status	No	822	154	1.0	1.0
	Yes	5	5	5(1.5, 18.7) *	4 (1.15, 16.5) *

**Note**: LRT [X^2^(11) = 54.63] and p < 0.000, Hosmer and Lemeshow Test = 9.585 (p = 0.295), Reference (ref.):1.0, *Variables statistically significant at p-value < 0.05

**Fig. 2:**
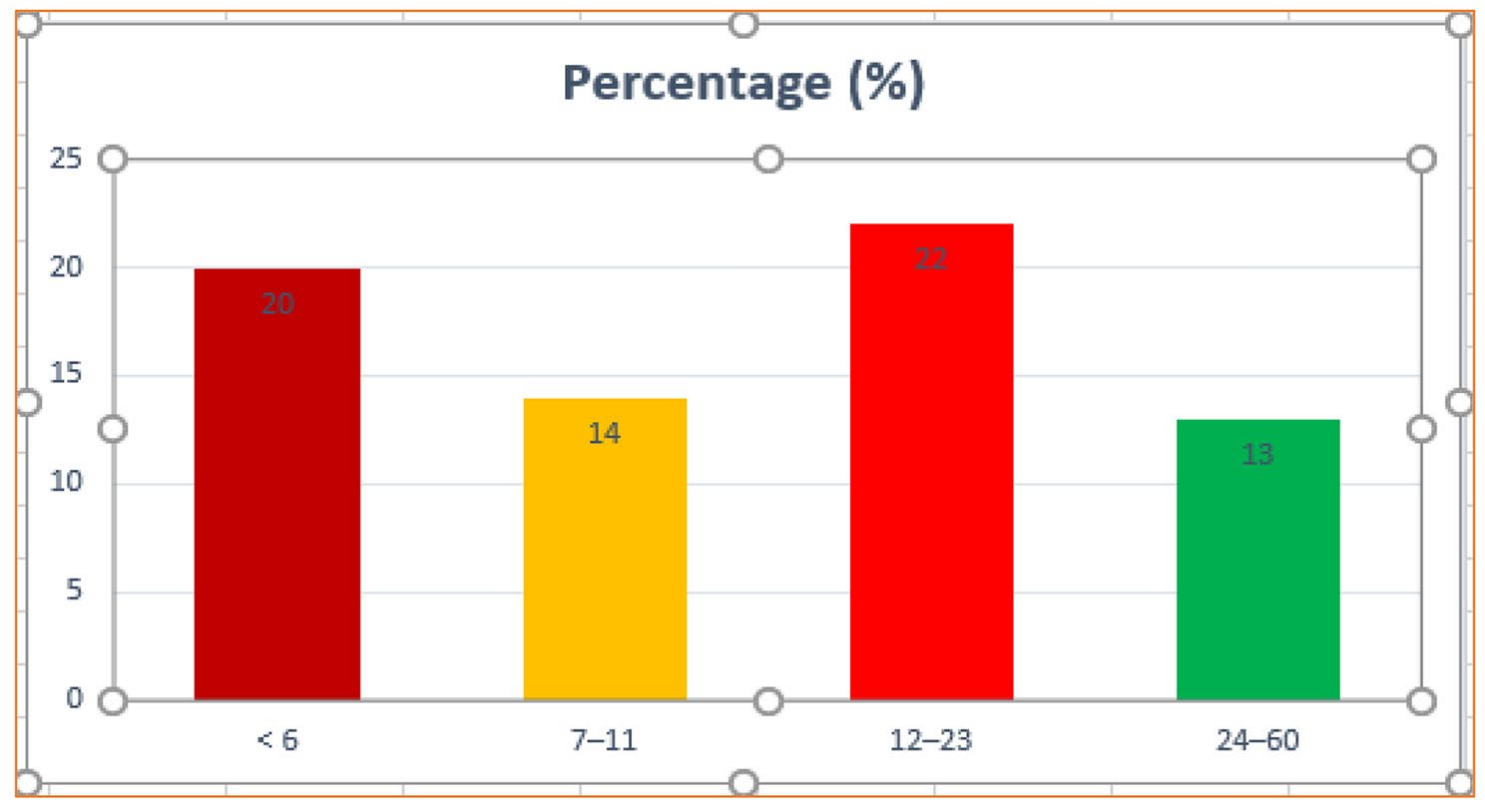
Prevalence of ARI among children under-five stratified by age in Tigray Regional State, northern Ethiopia, from January 18–June 27, 2016

The prevalence of ARI was highest in the age group 12–23 months (22%), followed by < 6 months (20%), and comparatively lower in the 24–60 months age group (13%) (Fig. 2).

### Predictor factors associated with ARI among children under-five years

In the multivariable logistic regression model analysis, the age of the child, wealth index, diarrhea status, and mother’s smoking cigarettes were significantly associated with ARI (Table 4). The results from multivariable logistic regression model analysis showed the odds of developing ARI among children in the age group 24–60 months were 59% times decreased (AOR: 0.59, 95%CI: 0.352–0.98) compared to the less than 6 months. Being the rich wealth index (AOR: 0.60, 95%CI: 0.378–0.959), diarrhea status of the child (AOR: 3, 95%CI: 1.97–4.73), and mother’s smoking cigarette (AOR: 4, 95CI: 1.15–16.50) were significant predictor factors of ARI (Table 4).

## Discussion

This study was conducted to assess the prevalence and predictor factors of ARI among children under-five years in the Tigray regional state, northern Ethiopia. The 2016 EDHS showed that ARI is still one of the leading causes of illness and mortality among children under five years. Both descriptive statistics and multivariable logistic regression analysis were used to analyze the secondary dataset. In the current analysis, the age of the child, wealth index of the household, diarrhea status of the child, and the mother’s smoking cigarettes were found to be significant predictor factors associated with ARI.

The result of this study showed that the prevalence of ARI among children under-five year was 16.10% [95%CI: 13.80–18.40]. This finding is in line with a systematic review done in Sub-Saharan African countries, 16% [18]. However, the result of this study was lower than the study conducted in the greater Accra region of Ghana, 3 L% [2], Northwest Ethiopia, 27.3% [1] and Bangladeshi, 21.3% [19].

Conversely, it was a higher burden than the survey report symptoms of ARI on 2016 EDHS conducted in Ethiopia, 6.6% [5], Zambia, 8% [20], Iraq, 6.9% [21] and rural Ethiopia, 7.8%.

Similarly, it was four times overburdened than the survey report on 2014 DHS conducted in Ghana, 4.0% [22] and Delhi slums, 4.5% [23]. The possible discrepancy might be due to differences in seasonal trends in ARI or discrepancies in the year and age of the study participants as well as the discrepancies in the study sites, study design and data collection.

The odds of ARI among children in the age group of 24–60 months were lower compared with those less than 6 months (AOR: 0.59, 95%CI: 0.35–0.98). This result was in line with the results of the study conducted in the greater Accra region of Ghana [2], rural Ethiopia [6], Ahmedabad city and other low- and middle-income nations [24, 25]. The possible explanation is that as a child grows older, he/she will have greater resistance to infectious diseases like cough and diarrhea [26, 27]. Since children’s immunity develops as they grow older, they are more prepared to fight against infection.

This study found that ARI was more common among diarrhea-infected children compared with non-diarrhea-infected children (AOR: 3, 95%CI: 1.97–4.73). The result is consistent with studies conducted in Ghana and Ethiopia [2, 6]. Moreover, studies were done in the Oromia zone, northeast Ethiopia [28], Zimbabwe [29], and southwest Ethiopia [30]. A cross-sectional study done in Bangladesh [31] also reported that children who had a history of diarrhea were at increased hazard for ARI. The possible explanation may be that children who have an illness like diarrhea may have a lowered immunity, making them more susceptible to an infection like ARI.

Children whose mothers smoked a cigarette were significantly associated with ARI among under-five children (AOR: 4, 95CI: 1.15–16.50). Mother’s smoking is a risk factor for several pulmonary infections; this may be due to its adverse effects on respiratory defenses. It is also associated with increased morbidity and mortality from pneumonia and influenza [32].

The wealth index was another significant socioeconomic predictor variable that affects ARI in Tigray Regional State. Children born from rich wealth index groups were founded, at a lower risk of ARI symptoms before celebrating their fifth birthday than those children from poor wealth index. However, this study contradicted another study conducted in Bangladesh [6, 33]. The probable explanation for the difference may be due to wealthier households tending to afford better nourishment and medical treatment for their children. This finding is consistent with other studies which found that higher poverty levels increase the risk of ARI and diarrhea [34, 35].

### Limitations of the study

This study identified the problem of ARI in the study area. However, some of the limitations ought to be taken into account. In this study due to the secondary retrospective nature of the data, the authors were unable to include some of the significant predictor variables which have been recognized as associated risk predictors due to the high rates of missing datasets for predictor variables. Due to this reason, the associated predictors studied were a convenience sample of the dataset collected as part of the DHS and it’s not clear how informative these results are (given the oversight of some of the historically important datasets). Moreover, the study was done 07 years back so it is unlikely to reflect the latest status of ARI in the region.

## Conclusions

The prevalence of ARI displays that Tigray regional state was experiencing a higher ARI rate than the national level. The current study identified the low wealth index of households, children aged (24–60 months), mothers smoking cigarettes, and diarrhea status of children as crucial predictor factors for ARI. Interventions should be improved to these modifiable major predictor factors that significantly decrease the ARI problem among under-five children.

According to the findings in this study, the following are recommended.

- Public health care providers in partnership with other stockholders should have planned to reduce the smoking status of mothers and diarrhea infection of Childs.
- The regional and federal Ministry of Health office should give attention to marking the mother’s familiarity concerning their health and Childs’ health when planning to control and prevent childhood diseases.

### Electronic supplementary material

Below is the link to the electronic supplementary material.

Supplementary Material 1

## Data Availability

Dataset is available online from www.measuredhs.com. A letter of approval for the use of the dataset was obtained from the Measure DHS and the data was downloaded from the website (https://dhsprogram.com/data/available-datasets.cfm). We used the 2016 EDHS children data and extracted the response and predictor variables.

## List of abbreviations

AOR: Adjusted Odds Ratio
ARI: Acute Respiratory Infection
CSA: Central Statistics Agency
CI: Confidence Interval
COR: Crude Odds Ratio
EDHS: Ethiopian Demographic Health Survey
IQR: InterQuartile Range
LRT: Log-Likelihood Ratio Test
VIF: Variance Inflation Factor
SSA: Sub-Saharan Africa
SD: Standard Deviation
