# Supplementary material for: Prevalence and predictors of acute respiratory infection among children under-five years in Tigray regional state, northern Ethiopia: a cross sectional study
Source: BMC Infect Dis. 2023 Oct 30;23:743. doi: 10.1186/s12879-023-08701-2 (PMC10614314; doi:10.1186/s12879-023-08701-2)
Supplement: Supplementary file 1 — Supplementary Material 1 [file 12879_2023_8701_MOESM1_ESM.docx]

Additional files

Additional file 1 Syntax in STATA version 14 for multivariable logistic regression model analysis.

Stata codes for data analysis.

Multivariable logistic regression analysis

/*All risk factors with p-value < 0.25 at the bi-variable logistic regression analysis were entered into the multi-variable logistic analysis to control confounding effects*/

logistic ARV i.Childs_age i.Sex_of_child i.Mothers_education i.Wealth_index i.Health_insurance_coverage i.Diarrhea_status i.Mothers_smoking_status i.Anemia_status_of_child

/*Multicollinearity test was used to assess the existence of correlation among covariates*/

vif, uncentered

/*Goodness-off- fit to the final model was checked by Hosmer and Lemeshow and LRT*/

estat gof

estat ic
